# Cardia Laxity under Retroflexed Endoscopy Is a Reflection of Esophageal Hiatus Enlargement

**DOI:** 10.1155/2020/9180167

**Published:** 2020-05-16

**Authors:** Dong Chen, Shurui Tian, Zhiwei Hu, Jimin Wu

**Affiliations:** Department of Gastroesophageal Surgery, PLA Rocket Force Characteristic Medical Center, Beijing, China

## Abstract

**Methods:**

Information from patients who underwent endoscopy and CT scan in our department was collected and analyzed retrospectively. Three-dimensional reconstruction of hiatus from CT images was performed using 3DSlicer software, and the degree of esophageal hiatus enlargement was compared with the degree of gastroesophageal laxity under retroflexed endoscopy.

**Results:**

Information from 104 patients was included for analysis. The Spearman correlation coefficient was 0.617 (*p* ≤ 0.001). When subgroup correlation analysis was performed according to the presence of hiatal hernia on CT, the Spearman correlation coefficient was 0.816 (*p* ≤ 0.001) in the hernia group and 0.351 (*p* = 0.002) in the nonhernia group. The proportion of hiatal hernia and severe esophagitis was increasing gradually with the degree of gastroesophageal laxity.

**Conclusion:**

The degree of gastroesophageal laxity (cardia or hiatus) under retroflexed endoscopy reflects the degree of esophageal hiatus enlargement; with the degree of gastroesophageal laxity increasing, the proportion of HH and severe esophagitis increases gradually. This may be useful for physicians in China to guide themselves in the selection of patients for endoscopic antireflux treatment.

## 1. Introduction

Gastroesophageal reflux disease (GERD) is a condition which develops when the reflux of stomach contents causes troublesome symptoms and/or complications [1, 2]. Among the objective methods for evaluation of GERD, pH-impedance monitoring and high-resolution manometry are not widely used in China, while almost every general hospital in China has endoscopy. In the field of GERD, it can not only identify esophagitis, Barrett's esophagus, and hiatal hernia but can also assess the degree of gastroesophageal laxity (cardia or hiatus) under retroflexed view. In clinical practice, we find that (1) the shape of most gastroesophageal laxity under retroflexed endoscopy is fusiform, which is corresponding to the shape of esophageal hiatus under laparoscopic view; (2) with the degree of gastroesophageal laxity under retroflexed endoscopy increasing, the component of laxity gradually shifted from cardia to esophageal hiatus; and (3) the degree of gastroesophageal laxity under retroflexed endoscopy is to some extent associated with the degree of esophageal hiatus enlargement under laparoscopic view. These observations indicated that gastroesophageal laxity under retroflexed endoscopy may be the refection of esophageal hiatus enlargement. For those showing as hiatus laxity under retroflexed endoscopy, only by observation can we know that the gastroesophageal laxity reflects the enlargement of esophageal hiatus, while for those showing as cardia laxity, we are not sure. The aim of this study was to confirm the correlation between gastroesophageal laxity (especially cardia laxity) under retroflexed endoscopy and objective enlargement of esophageal hiatus by data analysis.

## 2. Methods

### 2.1. Study Population

A retrospective analysis was performed on CT images and prospectively collected endoscopic information in our center between March 7, 2019, and December 8, 2019. Inclusion criteria (all) are as follows: underwent both endoscopy and CT scan within 1 month and the diaphragmatic crura can be distinguished from the surrounding tissues on CT images. Exclusion criteria (any one) are as follows: previous upper gastrointestinal surgery (include radio frequency therapy); CT slice thickness more than 2.5 mm; and inadequate range of CT scan for esophageal hiatus. The study was approved by the Ethics Committees of the PLA Rocket Force Characteristic Medical Center and registered together with the prospective part in the Chinese Clinical Trial Registry (ChiCTR1900025298), and the study conformed to the Declaration of Helsinki.

### 2.2. Endoscopy

**Figure 1:**
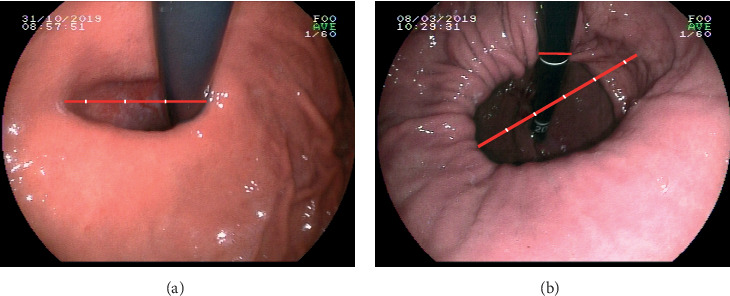
The image of maximum laxity with the long diameter: (a) at the horizontal level showing that the length was about 3.4 of an endoscope (perfect position); (b) not at the horizontal level showing the length of 5.3 of an endoscope (suboptimal position).

All the endoscopy was performed by one doctor with more than 10 years of experience, and images were prospectively collected since March 2019. During the test, after a routine examination of the esophagus, the body, the antrum, and the duodenum, retroflexed viewing of the fundus and gastroesophageal laxity was performed. Under retroflexed view, air inflation into the stomach was until the minimization of gastric folds; images of retroflexed view were obtained during circle observation; during this procedure, an increase in the degree of gastroesophageal laxity was observed. Considering that the degree of gastroesophageal laxity mainly relies on the size of a long diameter (a short diameter was slightly larger than the diameter of an endoscope in most cases) (Figure 1(a)), so we choose the long diameter as an indicator of gastroesophageal laxity. The images of maximum laxity were collected with the long diameter at the horizontal level as far as possible, so as to use the diameter of an endoscope as reference (about 1 cm) to estimate the length of the long diameter (Figure 1(a)) and eventually to assess the degree of gastroesophageal laxity under retroflexed endoscopy. The gastroesophageal junction (GEJ) was defined as the proximal end of gastric folds. Hiatal hernia (HH) was diagnosed when the GEJ is above the crural impression for ≥2 cm [3]. The presence of esophagitis was classified according to the Los Angeles classification [4].

### 2.3. 3D Reconstruction of Esophageal Hiatus

**Figure 2:**
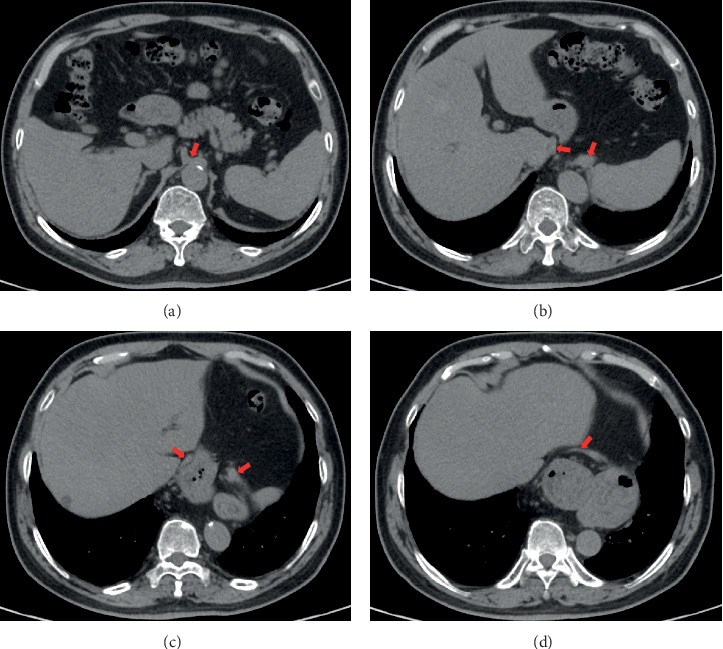
CT images showed the diaphragmatic crura from bottom to top: (a) the red arrow indicated the position where left and right crura began to separate; (b, c) the red arrow indicated the separated crus; (d) the red arrow indicated the left and right crus converge.

**Figure 3:**
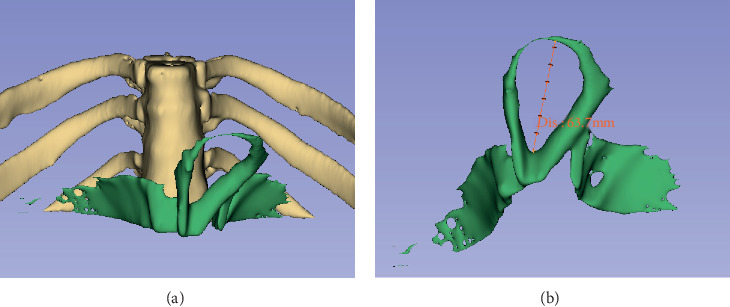
3D reconstruction of esophageal hiatus from CT images of Figure 2: (a) anteroposterior view of reconstructed vertebra, ribs, and esophageal hiatus; (b) objectively measured long diameter of esophageal hiatus, 63.7 mm.

3DSlicer is an open-source software platform for medical image informatics, image processing, and three-dimensional visualization. Its quantitative measurement of lesion dimensions had been validated and accepted by the U.S. FDA [5]. We performed 3D reconstruction of esophageal hiatus from CT images (Figure 2) using 3DSlicer (version 4.10.2) and objectively measured the long diameter of esophageal hiatus (Figure 3) independent of endoscopic data. In the meantime, HH was diagnosed when herniation of the stomach or any other celiac content through the hiatus into the chest was found on the CT images, and type III HH was diagnosed when the GEJ and stomach were located above the diaphragm. Patients were grouped according to the presence of HH on CT for subgroup correlation analysis.

### 2.4. Statistical Analysis

All statistical test was performed using IBM SPSS Statistics (version 24). The simple scatter diagram was used to evaluate monotone relation, The Spearman correlation coefficient was used to correlate the endoscope with the 3D model measured long diameter for both the whole cases and different groups according to the presence of HH on CT; statistical significance was considered at the *p* < 0.05 level.

## 3. Results

Between March 7, 2019, and December 8, 2019, 179 patients underwent both endoscopy and CT scan within 1 month, among which 75 patients were excluded for inadequate clear view of diaphragmatic crura on CT images; thus, information from 104 patients was included for analysis.

The mean age was 58.86 ± 11.95 years old, and male proportion was 57.7%. The simple scatter diagram showed a monotone relationship between CT and endoscopy-measured long diameter. The means of long diameter assessed by endoscopy was 2.6 (±1.1) cm vs. 30.3 (±12.4) mm by 3D reconstruction of esophageal hiatus based on CT images, and the Spearman correlation coefficient was 0.617 (*p* ≤ 0.001). When subgroup correlation analysis was performed according to the presence of HH on CT, the Spearman correlation coefficient was 0.816 (*p* ≤ 0.001) in the hernia group and 0.351 (*p* = 0.002) in the nonhernia group.

The proportion of HH on CT and endoscopy, together with type III HH and severe esophagitis, was increasing gradually with the degree of gastroesophageal laxity increasing (Table 1). When the long diameter was ≥3 cm, the proportion was already high for HH both on CT (62.5%) and on endoscopy (93.8%).

## 4. Discussion

By data analysis, we confirmed that gastroesophageal laxity under retroflexed endoscopy is positively correlated with the enlargement of esophageal hiatus, no matter as a whole or as subgroups according to the presence of HH on CT. We also found that the proportion of HH and severe esophagitis increases according to the degree of gastroesophageal laxity.

As for the method used for evaluating gastroesophageal laxity, Seltman et al. [6] used a software to measure the circumference of the endoscope at the position when it passes through the cardia, so as to calculate the circumference of a circle that covers the relaxed area of the cardia, while in our experience, the relaxed gastroesophageal area was not circular in most cases, and the circumference changes significantly during endoscopy. In addition, the impact of deformed images was not taken into consideration, so the method in this study was not suitable for us. Ihde et al. [7] applied a 1 cm diameter of the endoscope as a reference to estimate the long diameter of the relaxed gastroesophageal area. This method is more reasonable than the previous one, in that the largest long diameter cannot be difficultly obtained during endoscopy, but the way to measure the diameter of endoscope was suboptimal, and the fact that the images from endoscopy were deformed could not be neglected. As we could see from Figure 1, the endoscope was larger on the upper position; the underlining reason may be the difference in the distance from the light source. As a result, we put the long diameter at the horizontal position, so as to better choose the diameter of the endoscope as a reference and minimize the impact of deformed images.

As for the objectively measured esophageal hiatus, we first thought about using data measured during antireflux surgery, but considering that in patients undergoing antireflux surgery, the degree of gastroesophageal laxity under retroflexed endoscopy is generally high and the data of patients with low degree of laxity (which contain more cardia laxity) may be difficult to collect, so we choose an alternative way. Three-dimensional reconstruction is popular in recent years, and many software can be used, among which 3DSlicer is a free software for three-dimensional visualization of medical images, which had been widely used in neurosurgery and the respiratory system [8–12]. We used 3DSlicer to reconstruct the esophageal hiatus and objectively measured the long diameter. By comparison, we confirmed that the degree of gastroesophageal laxity under retroflexed endoscopy is positively correlated with the enlargement of esophageal hiatus (0.617, *p* ≤ 0.001), which is consistent with our clinical observations. After confirming the positive correlation as a whole, we still need to confirm it in subgroups. As the diagnosis HH on CT is mainly based on the presence of an hernia sac, therefore, subgroups (HH on CT or not) match well with cardia or hiatus laxity under retroflexed endoscopy; as we can see from the result parts, the Spearman correlation coefficient was 0.816 (*p* ≤ 0.001) in the hernia group and 0.351 (*p* = 0.002) in the control group, which confirmed our observation that the degree of gastroesophageal laxity (cardia and hiatus) actually reflects the degree of esophageal hiatus enlargement.

As we all know, antireflux surgery is widely performed in western countries, but in China, this filed is at a starting stage. Most patients with GERD symptoms in China are visiting the department of digestive internal medicine; thus, the physicians have a critical role in the screening, diagnosis, and treatment distribution of GERD patients. While they are only allowed to perform endoscopic antireflux treatment, they sometimes face the dilemma of performing endoscopic treatment or referring patients to surgical treatment when the indications for intervention are fulfilled by symptoms, pH-impedance monitoring, and/or endoscopy. In this circumstance, HH on endoscopy is usually not present, as clinical experience suggests that the more relaxed the gastroesophageal zone, the higher the failure rate of endoscopic treatment and the higher the necessity for surgical treatment; thus, the degree of gastroesophageal laxity is under full consideration for choice of treatment options. In this study, we confirmed that the degree of gastroesophageal laxity is positively correlated with objective enlargement of esophageal hiatus, which is consistent with their clinical experience, in that endoscopic treatment could not handle the problem of esophageal hiatus enlargement, and the higher the degree of esophageal hiatus enlargement, the higher the necessity for surgery. Another finding of this study is that the proportion of HH and severe esophagitis increases gradually with the degree of gastroesophageal laxity under retroflexed endoscopy, and when the long diameter ≥ 3 cm but <4 cm, the proportion of HH on CT and endoscopy has already reached up to 62.5% and 93.8%, respectively, which further suggested a gradually clear indication and necessity for antireflux surgery. In addition, we provided a simple and practical way to assess the degree of gastroesophageal laxity; thus, the physician could use this simple way as reference of hiatus enlargement and guide themselves in the selection of patients for endoscopic antireflux treatment, but further studies with follow-up data are needed to detail the cutoff value for patient selection.

In summary, the degree of gastroesophageal laxity (cardia or hiatus) under retroflexed endoscopy reflects the objective enlargement of esophageal hiatus; with the degree of gastroesophageal laxity increasing, the proportion of HH and severe esophagitis increases gradually. This may be useful for physician in China to guide themselves in the selection of patients for endoscopic antireflux treatment, while further studies are needed to illustrate the cutoff value for patient selection.

## Figures and Tables

**Table 1:** HH and esophagitis according to different degrees of gastroesophageal laxity.

Long diameter (cm)	>1, <2	≥2, <3	≥3, <4	≥4, <5	≥5
N	22	56	16	5	5
HH on CT	9.1%	14.3%	62.5%	80%	100%
Type III HH	0%	0%	12.5%	40%	100%
HH on endoscopy	13.6%	42.9%	93.8%	100%	100%
Esophagitis	22.7%	51.8%	62.5%	40%	60%
LA-A	4.5%	12.5%	0%	0%	0%
LA-B	13.6%	32.1%	43.8%	20%	0%
LA-C	4.5%	7.1%	18.8%	0%	40%
LA-D	0%	0%	0%	20%	20%

LA: Los Angeles; HH: hiatal hernia.

## Data Availability

Data without patients privacy information will be available by contacting the corresponding author after publication of this paper.
